# Is the Internet a useful and relevant source for health and health care information retrieval for German cardiothoracic patients? First results from a prospective survey among 255 Patients at a German cardiothoracic surgical clinic

**DOI:** 10.1186/1749-8090-1-36

**Published:** 2006-10-21

**Authors:** Dietrich Stoevesandt, Claudius Diez

**Affiliations:** 1 Department of Radiology, Martin-Luther-University Halle-Wittenberg, Ernst-Grube-Str. 40, D-06097 Halle/Saale, Germany; 2 Department of Cardiothoracic Surgery, Martin-Luther-University Halle-Wittenberg, Ernst-Grube-Str. 40, D-06097 Halle/Saale, Germany

## Abstract

**Background:**

It is not clear how prevalent Internet use among cardiopathic patients in Germany is and what impact it has on the health care utilisation. We measured the extent of Internet use among cardiopathic patients and examined the effects that Internet use has on users' knowledge about their cardiac disease, health care matters and their use of the health care system.

**Methods:**

We conducted a prospective survey among 255 cardiopathic patients at a German university hospital.

**Results:**

Forty seven respondents (18 %) used the internet and 8,8 % (n = 23) went online more than 20 hours per month. The most frequent reason for not using the internet was disinterest (52,3 %). Fourteen patients (5,4 %) searched for specific disease-related information and valued the retrieved information on an analogous scale (1 = not relevant, 5 = very relevant) on median with 4,0. Internet use is age and education dependent. Only 36 (14,1 %) respondents found the internet useful, whereas the vast majority would not use it. Electronic scheduling for ambulatory visits or postoperative telemedical monitoring were rather disapproved.

**Conclusion:**

We conclude that Internet use is infrequent among our study population and the search for relevant health and disease related information is not well established.

## Background

Several international studies suggested that more than half and as much as 80 % of adults with Internet access use it for health care purposes [4,10,13]. These estimates have been widely distributed and now frequently form the context for discussions among the media and other of the role of the Internet in health care. Other, less well-publicized reports suggest much lower rates of Internet use [2,9]. In fact, little is known about to what extent patients in different countries access the Internet and whether they benefit from consulting it [5,8].

Cardiovascular diseases are the leading cause of mortality in the industrialized world and interventional or operative procedures have been increasingly used to prevent cardiac damage. Recent innovations in cardiac surgery, e.g. minimally invasive or off-pump procedures, have significantly decreased morbidity and mortality rates among cardiopathic patients [6,17]. An increasing number of patients now undergo coronary surgery without cardiac arrest and the heart-lung-machine.

Despite the recent progression and the new achievements in this field, patients still remain to be informed about the indications, risks, benefits and outcome of cardiac surgery [11,14]. Unfortunately, the majority of cardiopathic patients and their families remain unaware of new innovative surgical techniques. Adequate information on the Internet could increase the patient's knowledge about these procedures, their applications and limitations [3,12]. We therefore focused our study at a midsize German cardiothoracic clinic in former Eastern Germany on three central research questions: Do cardiopathic patients and their families access the Internet to obtain health-related information? If this is the case, is online medical information understandable and useful for this non-medical public? Are there demographic and socioeconomic differences among internet and non-internet users?

## Methods

### Study sample

A prospective study among 255 patients, who underwent elective cardiac and thoracic operations was conducted between December 2003 and February 2004 at the Department of Cardiothoracic Surgery at the University Medical Centre Halle. Emergency patients and patients > 85 years were excluded from the study. A questionnaire comprising 25 questions was answered by the patients on the day of admission in the presence of a final year medical student. The student explained the questions and, if necessary, and the different meanings of the answers.

### Statistical analysis

Data were collected in an Excel sheet. Discrete and continuous variables were analysed using the Chi-Square test, Fisher's exact test (where appropriate) or the Student t-test. Differences between variables were considered significant when p < 0,05. All calculations were performed with SPSS 11.0 [15] and StatsDirect 2.0.3 [16]. Data are presented either as mean ± standard deviation or, where appropriate, as median and the 25^th ^and 75^th ^percentile, respectively.

## Results

### Demographic data

Two hundred fifty-five patients who met the inclusion criteria constituted the final sample. Mean age of the participants was 62,98 ± 11,56 years. Table 1 describes the sociodemographic characteristics of the sample more detailed. More than two-thirds of our participants were still married (n = 190, 73,1 %), twenty were divorced (7,7 %), seventeen were single (7,5 %) and twenty-four were widowed (9,2 %). On average, every patient lived with 2,11 ± 0,88 relatives at home (median 2,0).

**Table 1:** Sociodemographic characteristics of survey sample (n = 255)

	n (%)
	
*Age (years)*	
20 – 49	26 (10,2)
50 – 64	94 (36,8)
65 – 80	132 (51,8)
> 80	3 (1,2)
	
*Gender*	
female	69 (27)
male	186 (73)
	
*Education*	
no school	5 (2)
8^th ^– class	132 (52)
10^th ^– class	65 (25,5)
A-levels	9 (3,5)
university degree	44 (17)
	
*Profession*	
Senior citizen	182 (71)
employees and manager	30 (12)
self-employed person	10 (4)
Student	2 (0,8)
worker	3 (1,2)
unemployed	28 (11)

A-levels usually refers to the completion of twelve school years, which is prerequisite for attending a university in Germany. As males are affected more extensively by cardiovascular diseases than woman, hence our study population included more than twice the number of males than females.

Considering Halle as a major German city with more than 220000 inhabitants, only 63 patients (24,2 %) came from Halle, the remaining lived in smaller cities and non-urban areas around Halle (n = 192, 75,8 %). Table 2 summarizes the reasons for admission at our clinic.

**Table 2:** Operation among the survey sample (n = 255)

	n (%)
	
Coronary artery bypass grafting (CABG)	158 (60,8)
Pacemaker/Defibrillator implantation	16 (6,2)
Cardiac valve replacement/reconstruction	16 (6,2)
Lung operation	15 (5,8)
Combination (CABG and valve)	23 (8,5)
Congenital defect repair	1 (0,4)
Other	26 (10)

### Prevalence and frequency of internet use

Almost 70 % of our participants did not own a personal computer (n = 179), whereas twenty-one (8,1 %) had a PC, but no internet access, and fifty-five (20,8 %) had a computer with internet access. Interestingly, only 18 % (n = 47) used the internet, the remaining participants had never used the internet before. Thirty-six (13,8 % of total) used the internet at home, seven (2,7 %) accessed it at the working place and four (1,6 %) went online in an internet café (n = 1) or at friends/family (n = 3). Among the internet users, there were six women and 41 men (53,5 ± 14,2 versus 49,7 ± 17,8 years, p = n.s.). There were no significant differences between female and male users with respect to education, duration of internet use and computer possession.

Considering the monthly internet use, twenty-three (8,8 %) internet users accessed the web more than 20 hours per month, eight (3,1 %) used it 10 – 20 hours per month, eight 5 – 10 hours per month and the remaining eight participants went online less than 5 hours per month. We examined the reasons why and how often patients used the internet (Table 3). Only 17 respondents used the internet for health or health care information without a gender preference (2/6 women versus 15/41 men, p = n.s.).

**Table 3:** Prevalence and Frequency of Internet Use (n = 47 internet users)

*How often did you*	*daily*	*1 – 2/week*	*1 – 2/month*	*1 – 2/3 month*
use the internet for email (n = 34)	17	14	2	1
use the internet for retrieving general information (n = 47)	20	23	3	1
use the internet for information about health or health care (n = 17)	1	6	7	3
use the internet for amusement (n = 29)	4	11	10	4
use the Internet for shopping (n = 26)	2	4	8	12
use the internet for chatting (n = 16)	1	3	8	4
use the internet to communicate with other people in newsgroups (n = 5)	0	3	1	1
use the internet to maintain your own homepage (n = 4)	0	1	2	1

The search for health or health care information has not been well established yet among our study sample.

The reasons for not using the internet were as follows: disinterest (n = 136, 52,3 %), too complicate (n = 24, 9,2 %), too expensive (n = 21, 8,1 %), don't know (n = 3, 1,2 %).

### Internet use and retrieval of relevant health information

We also asked the patients whether or not they looked for information about her/his specific cardiac/thoracic disease and the planned operation at our hospital. Only 14 respondents (5,4 %) reported to have used the internet for retrieving specific information about cardiac or thoracic diseases. Interestingly, but not significantly, no female patients searched for specific disease information, whereas fourteen male patients went online to look for specific information (p = 0,19). Additionally, the families or friends of 12 patients went online to find information on the specific conditions. Asked how relevant these information had been, respondents rated them on an analogous scale from 1 (not relevant) to 5 (very relevant) on median with 4,00 (range 3 – 4,5). Nine of those who used the internet for health or health care information retrieval said, that internet information helped to better understand their symptoms and their planned treatment. Only one respondent found the information helpful to manage the own condition.

As cardiovascular diseases comprise the most popular and most cost-intensive disorders, we wanted to know, if patients use specific newsgroups or online chats. Only one internet user had ever tried to use these specific offer to find relevant information.

### Internet use is age and education dependent

Internet users in our sample were significant younger than non-user (median 57 (60, 72) versus 67 (47, 63) years, p < 0,0001). Moreover, internet user had a higher education level. Nineteen of 47 users (40,4 %) had a university degree, 12,76 % (n = 6) had the A-levels and 16 patients (34 %) finished the 10^th ^class of a secondary school. The majority of non-users (n = 126, 60,57 %) had finished the 8^th ^class of a secondary school, 49 patients (23,55 %) finished the 10^th ^class of a secondary school, 5 did not attend any school, whereas only 12 % (n = 25) had a university degree.

### Impact of a specific web-based interface of our department

As our clinic maintains an own website containing relevant information for our patients (e.g. information about specific operation procedures), we wanted to know, if and how our patients value electronic information technologies.

Asked if the patients had ever visited our department website, only five (1,96 %) respondents agreed, whereas the vast majority disagreed. As we plan to extend our electronic patient service, we asked how special services would be evaluated. First, we asked if an extended drug information service, e.g. a detailed explanation of certain side and cross effects of drugs prescribed after surgery, would be useful. More than 75 % of all respondents found such an offer not useful and irrelevant (n = 216, 85 %) and only 39 patients (15 %) would use such a service. Considering a postoperative intermittent telemedical monitoring of some basic cardiac functions, e.g. heart rhythm, four out of five patients (n = 204, 78,5 %) would not participate in such a service. Even electronic pre- and postoperative scheduling for ambulant visits have not positively valued. Almost 90 % (n = 224) of our patients preferred phone calls and information letters to an email.

Asked which health/disease related themes might be worth an electronic information delivery by email or special sections on our website, 189 participants had no interest, whereas the small remaining minority would liked to be informed on several topics such as postoperative physical and sexual behaviour or advice for emergencies.

Sixty-three (24,2 %) of all respondents would use the web-based resource, 95 patients (36, 5 %) would only use the already existing sources of information (e.g. physicians, pharmacies) and the remaining 97 respondents interestingly (37,5 %) found a printed brochure much more useful than any electronic resource.

In summary, our respondents valued electronic information on an analogous scale from 1 (not useful) to 5 (very useful) on median with 1,0. Asked if they would now visit our clinic website after answering the questionnaire, still 73,5 % (n = 191) would not and only 63 patients (24,2 %) might visit our site to find useful information.

## Discussion

Current estimates suggest that an increasing number of people access the World Wide Web and at least 2 % out of more than 3 billion websites are assessed to be health related [1,10]. However, many patients that could actually benefit from proper use of the World Wide Web are unfamiliar with the new computer technologies and in the majority of cases are not even internet users. A recent report from the German Federal Statistic Office showed that Internet use varies widely within Europe, North America and Asia and represents a highly dynamic market with rapidly growing numbers of users. In 2002, 44 % of German households had internet access, whereas e.g. 66 % of all Dutch households and 51 % of all US-households were equipped with Internet access [18]. In Spain and Japan, in contrast, 30 % and 35 %, respectively, of all households had Internet access. Only 21 % of patients in our sample possessed a PC with internet access and only 18 % of respondents used the Internet regularly. Internet use is age, income and education dependent [1] and our data are consistent with these findings. The older people are, the less frequent the internet access is. Forty-one percent of all Germans between 55 – 64 years have a PC with internet access, whereas this number decreases to 20 % in the group of all 65 – 74 years old Germans and drops to 12 % in the group of the 75 years and older Germans. Having a personal computer with internet access does not imply that the internet is used regularly. Only 8 % of all Germans older than 65 years and 27 % between 55 – 64 years used the internet in 2003. Among the European Community, internet use in Germany ranks far behind Scandinavia and Great Britain [18].

The reasons for the low PC and internet use in our study sample below the German and European average might be due to differences in the social structure between Western- and Eastern Germany such as the higher unemployed rate and the higher percentage of retired people and a less frequent internet usage in these groups compared to employees and students [18]. Furthermore, the internet users in our sample were significantly more educated than non-users and achieved higher academic degrees.

Although an increasing number of health-related websites became accessible, only 14 patients in our sample used this service. Health information retrieval played only a minor role for the internet users in our sample population. A recently conducted survey among German households showed that 24 % of Eastern German internet users search for health information online, in contrast to 35 % of Western Germans. The reasons for this difference have not been elucidated yet. One possible reason might be the latent fear in former Eastern Germany for potential misinformation and incredibility present in unprofessional websites and the underdeveloped contact to websites developed by the immediate health-care provider. We also believe that there is a growing need for objective, reproducible, widely accepted criteria that can form the basis for regulation of publication of medical material on the internet [7].

The interest in an extensive health-related internet use of our study sample is quite low. Although those, who went online for health information retrieval, valued the received information positively, other resources such as newsgroups, self-help groups, chat rooms and even visiting our clinic homepage, where several additional services are offered, have been fairly unknown among our study sample. There is a clear demand for continuing education, especially for senior people, to facilitate the access to the growing, modern and indispensable medium internet. Despite all the advantages the internet offers, printed media and personal contacts still proved to be the most reliable source of information among our study sample.

### Study limitations

This study is limited to one medium-sized Eastern German university with its specific demographic and social conditions. Since Halle with its strong industrial background before reunification and nowadays a high rate of unemployment and retired people represents a typical former Eastern German industrial city, we probably underestimate the real prevalence and frequency of internet use in our study compared to other areas in Western Germany.

## Conclusion

Continuing education, particularly for the elderly, remains necessary to facilitate the computer and internet use in former Eastern Germany. The advantages of electronic web-based media and professional health-care websites have not been fully employed by our sample yet. Conversely, web-based health care resources would be used more extensively, if adequate information was available to the patients.

## Competing interests

The author(s) declare that they have no competing interests.

## Authors' contributions

DS examined and selected the study patients. He explained the questionnaire to the patients and collected the data in a database. He also was involved in the coordination and interpretation of the data.

CD designed the study, developed the questionnaire, performed the statistical analysis and drafted the manuscript and revised it in cooperation with the co-author.
